# Macrophages Down-Regulate Gene Expression of Intervertebral Disc Degenerative Markers Under a Pro-inflammatory Microenvironment

**DOI:** 10.3389/fimmu.2019.01508

**Published:** 2019-07-03

**Authors:** Ana J. Silva, Joana R. Ferreira, Carla Cunha, João V. Corte-Real, Mafalda Bessa-Gonçalves, Mario A. Barbosa, Susana G. Santos, Raquel M. Gonçalves

**Affiliations:** ^1^i3S – Instituto de Investigação e Inovação em Saúde, Porto, Portugal; ^2^INEB – Instituto de Engenharia Biomédica, Porto, Portugal; ^3^ICBAS – Instituto de Ciências Biomédicas Abel Salazar, Universidade do Porto, Porto, Portugal; ^4^FCUP – Faculdade de Ciências da Universidade do Porto, Porto, Portugal

**Keywords:** intervertebal disc, inflammation, tissue regeneration, organ culture, *ex vivo* model

## Abstract

Low back pain is a highly prevalent clinical problem and intervertebral disc (IVD) degeneration is now accepted as the major pathophysiological mechanism responsible for this condition. Accumulating evidence suggests that inflammation plays a crucial role in the progression of human IVD degeneration, with macrophages being pointed as the key immune cell players in this process since their infiltration in degenerated IVD samples has been extensively demonstrated. Since they are highly plastic, macrophages can play different roles depending on the microenvironmental cues. The study of inflammation associated with IVD degeneration has been somehow neglected and one of the reasons is related with lack of adequate models. To overcome this, we established and characterized a new model of IVD organ culture under pro-inflammatory conditions to further dissect the role of macrophages in IVD associated immune response. For that, human monocyte-derived macrophages were co-cultured either with bovine caudal IVD punches in the presence of the pro-inflammatory cytokine IL-1β, or IVD-conditioned medium (CM), to investigate how IVD-produced factors influence macrophage phenotype. After 72 h, metabolic activity, gene expression and cytokine profile of macrophages and IVD cells were measured. Our results show that macrophages and IVDs remain metabolically active in the presence of IL-1β, significantly upregulate CCR7 gene expression and increase production of IL-6 on macrophages. When treating macrophages with IL-1β-IVD-CM, CCR7 upregulation follows the same trend, while for IL-6 an opposite effect was observed. On the other hand, macrophages interfere with IVD ECM remodeling, decreasing MMP3 expression and downregulating aggrecan and collagen II gene expression in the presence of IL-1β. Overall, the co-culture model established in this study can be considered a suitable approach to address the cellular and molecular pathways that regulate macrophage-IVD crosstalk, suggesting that degenerated IVD tissue tends to polarize human macrophages toward a more pro-inflammatory profile, which seems to aggravate IVD degeneration. This model could be used to improve the knowledge of the mechanisms that link IVD degeneration and the immune response.

## Introduction

Low back pain (LBP) is a common clinical problem affecting about 70–85% of the world population (1). The efficacy of the current clinical solutions is limited by our lack of understanding of the LBP pathomechanism, however it is accepted that the pain associated with intervertebral disc (IVD) degeneration, without evident signs of nerve compression, is the main cause of chronic LBP (40% of the cases) (2). Although IVD degeneration is a complex and multifactorial process, it is known to involve the loss of proteoglycans and water content in nucleus pulposus (NP), the central gelatinous tissue of IVD, with up-regulation of metalloproteinases (MMPs) and inflammatory mediators (3). These molecules can be produced by both IVD cells or immune cells, such as macrophages (4–6). Macrophages were identified in human herniated IVD samples in several studies, associated with increased disc degeneration (5, 7–9), but they are also suggested to have an important role in the spontaneous hernia regression, a rare event occurring in some LBP patients (10). These immune cells are also implicated in non-herniated IVD degeneration, although this topic remains poorly understood (5, 11–13). Recent studies demonstrated that macrophages are the only type of inflammatory cells infiltrated in the degenerated NP tissue and this is correlated with disease progression (12). These results are in accordance with the conclusions of Nakazawa et al. but specifically in non-herniated discs (13). Thus, on one side macrophages have been associated with a higher inflammatory response and increased levels of IVD degeneration, while on the other side, they have also been linked to the phenomenon of spontaneous hernia regression. These apparent controversial results can be explained by the high plasticity of macrophages, that can express different functional profiles in response to distinct environmental cues, from the classic pro-inflammatory M1 to a more pro-regenerative M2 phenotype.

It is clear that the inflammatory microenvironment created by macrophages and IVD cells plays an important role during IVD degeneration. However, there is a need to deepen the knowledge of the mechanisms that link degeneration of IVD and the immune response.

There is a lack of adequate models to study inflammation within IVD degeneration. Most of the *in vitro* studies conducted so far rely on 2D co-cultures of IVD or NP cells and macrophages. Most of the 2D culture systems do not mimic the harsh, hypoxic and degenerative IVD 3D microenvironment and impair IVD cells to produce native IVD extracellular matrix (ECM) (14). Additionally, the currently used *in vivo* models do not mimic the natural process of human IVD degeneration. Thus, *ex vivo* organ culture models not only allow the study of IVD degeneration in a more physiologically relevant environment than cell models, but also reduce the costs and ethical issues of *in vivo* experiments (15). For explant experiments IVDs can be isolated from different species. Although IVDs should ideally be isolated from human tissue, this material is difficult to obtain because of ethical and government regulatory restrictions and it is not very abundant. Alternatively, bovine IVD has been proposed as a suitable biological and biomechanical model for studying human disc disorders, since it is easily available and shows high similarities with human samples in terms of size, mechanical loading, composition, cell phenotype and distribution (15, 16). Our group has previously established an *ex vivo* proinflammatory/degenerative IVD organ culture model to be used as a more physiological model for drug and cell therapies screening (17). This model was successfully used to study anti-inflammatory nanoparticles (18) and the regenerative and immunomodulatory role of mesenchymal stem cells in IVD (19).

The models of IVD degeneration used so far often lack the presence of macrophages and if present, they are usually derived from mouse or immortalized cell lines, which are not completely representative of human macrophages since they have different gene expression profiles (20). By using monocyte-derived macrophages from human peripheral blood in this study, we adopted a more accurate model to study macrophage polarization *in vitro*, but in a context that resembles the *in vivo* conditions.

In the current work, we propose to study the macrophage crosstalk with IVD in a 3D IVD organ culture with associated human macrophage/immune response in pro-inflammatory conditions.

## Materials and Methods

### Human Primary Monocyte Isolation and Differentiation

Human primary monocytes were obtained from buffy coats of healthy blood donors, after informed consent and ethical approval of Centro Hospitalar S. João, as previously described by Oliveira et al. (21). Briefly, buffy coats were centrifuged at room temperature (RT) for 20 min at 1,200 g, without active acceleration or brake, for blood components separation. Peripheral blood mononuclear cell (PBMC) layer was collected and incubated with RosetteSep human monocyte enrichment isolation kit (StemCell Technologies) for 20 min, under gentle mixing, according to the manufacturer's instructions. The mixture was then diluted at a 1:1 ratio with 2% fetal bovine serum (FBS, Biowest) in phosphate buffered saline (PBS), gently layered over Histopaque-1077 (Sigma) and centrifuged as described above. The enriched monocyte layer was collected, and washed with PBS for platelet depletion, by centrifugation at 97 g for 17 min. Recovered monocytes were seeded on 6-well transwell cell culture inserts (Corning, Cat. No. 353102) at a density of 5 × 10^5^ cells/transwell. For monocyte-macrophage differentiation, cells were cultured in RPMI1640 medium (with GlutaMax) (Invitrogen) supplemented with 10% FBS (Biowest), 1% penicillin-streptomycin (P/S, Invitrogen) (macrophage culture medium), in the presence of 50 ng/mL of macrophage colony-stimulating factor (M-CSF, Immunotools). Cells were maintained in a humidified incubator, at 37°C and 5% CO_2_. After 7 days, cell culture medium was replaced without M-CSF renewal.

### Bovine IVD Tissue Isolation and Culture

Bovine IVD tissue was isolated from tails of young animals (~12 months old) from a local slaughterhouse, immediately after animals sacrifice, accordingly with ethical approval from the national veterinary authorities. Caudal discs were isolated and cultured as previously described (17). Briefly, standardized disc punches (with diameter of 9 mm) were collected with NP in the center and few surrounding annulus fibrosus (AF) and maintained for 4 days in 6-well tissue culture plates, with transwell cell culture inserts and 0.46 MPa static loading. The constraining effect on IVDs organ cultures has been previously described (17) and the weight used for simulation of static loading was optimized to corresponds to physiological loads during standing phase (22). IVDs were cultured in Dulbecco's Modified Eagle's Medium with low glucose (DMEM, Invitrogen), supplemented with 5% FBS (Biowest), 1% penicillin/streptomycin (Invitrogen), 0.5% fungizone (Invitrogen) and with the osmolarity adjusted to IVD-physiological 400 mOsm by addition of 1.5% of a 5 M NaCl/0.4 M KCl solution (IVD culture medium) (0.030 ± 0.007 ml/mg tissue). Samples were incubated at reduced oxygen atmosphere (37°C, 6% O_2_ and 8.5% CO_2_) and saturated humidity. Culture medium was replaced on the day after IVD isolation. In order to create a proinflammatory environment in some conditions, 4 days after IVD isolation, IVDs were needle-punctured (21G), medium was renewed (5 ml) and supplemented with 10 ng/mL of recombinant human IL-1β (PeproTech), and 3 h later, the co-culture with macrophages was performed. After the proinflammatory stimuli some of the discs were left in culture for additional 2 days in order to produce IVD conditioned media (IVD-CM). IVD-CM were centrifuged at 900 g for 5 min at 4°C and stored at −20°C until use.

### Establishment of Macrophage-IVD Co-cultures

**Figure 1:**
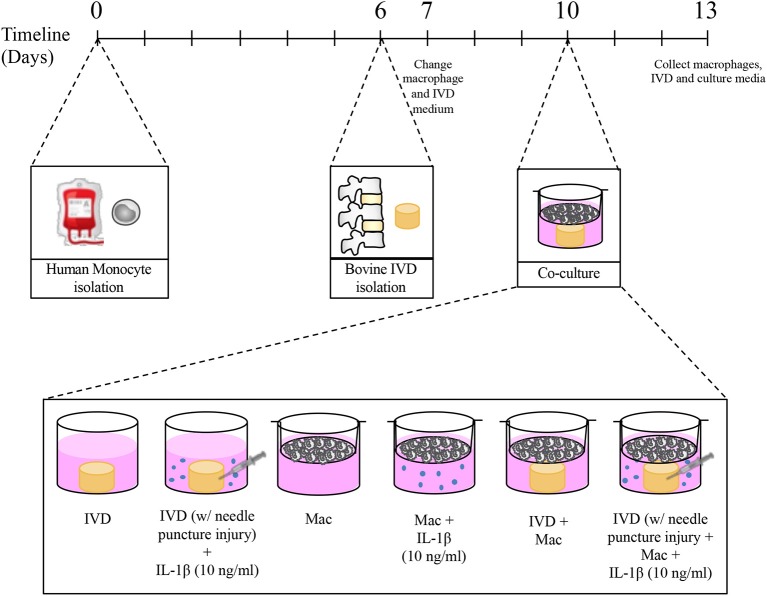
Experimental timeline and different culture conditions.

Ten days after monocyte isolation and 4 days after IVD isolation the transwells containing macrophages were transferred to the plates where IVDs were previously cultured. The permeable PET membrane of 1 μm pore size avoided macrophages to cross from the top to the lower compartment, allowing however the exchange of soluble factors between macrophages and IVDs. The co-cultures were maintained for 3 days in 5 ml of culture medium in a 1:1 proportion of macrophage medium and IVD medium and incubated at reduced oxygen atmosphere (37°C, 6% O_2_ and 8.5% CO_2_) and saturated humidity. Some conditions were supplemented with 10 ng/mL IL-1β. The experimental setup and respective conditions are schematically presented in Figure 1.

### Macrophage Treatment With IVD-CM

Ten days after monocyte isolation macrophages were cultured in 5 ml of culture medium a 1:1 proportion of macrophage culture medium and IVD-CM, respecting the same volume/cells ratio that in macrophage-IVD co-cultures, and incubated at reduced oxygen atmosphere (37°C, 6% O_2_ and 8.5% CO_2_) and saturated humidity for additional 3 days.

### Macrophage and IVD Metabolic Activity

The IVD and macrophage metabolic activity was determined through resazurin reduction assay. Briefly, after macrophage-IVD co-culture, the transwells containing macrophages were transferred to another plate. Both, IVDs and macrophages were separately incubated with resazurin redox dye (0.01 mg/mL) (Sigma-Aldrich) for 4 h at reduced oxygen atmosphere (37°C, 6% O_2_ and 8.5% CO_2_) and saturated humidity. Fluorescence intensity was measured (530 nm Ex/590 nm Em) using the multi-mode microplate reader Synergy MX (BioTek). Data is presented in percentage relative to control macrophages or control IVD.

### Macrophage Surface Marker Expression

Macrophages were incubated with PBS-EDTA at room temperature during 20 min and harvested by gently scrapping. Cells were washed and resuspended in FACS buffer (PBS, 2% FBS, 0.01% sodium azide) containing appropriate conjugated antibodies, and stained in the dark for 30 min at 4°C. Macrophages were immunostained with the following antibodies: anti-human CD14-APC (clone MEM-18), CD86-FITC (clone BU63) (both immunotools) and CD163-PE (clone GHI/61) (R&D Systems). To define background staining isotype matched antibodies were used as negative controls. After additional washing steps, cells were acquired on a FACS Canto Flow Cytometer (BD Biosciences) with BD FACSDiva software. Results were analyzed using FlowJo software version 10 (TreeStar, Inc.).

### Total RNA Isolation and Reverse Transcription for qPCR

Total RNA was isolated from macrophages and IVD cells using TRIzol reagent (Invitrogen) following manufacturer's instructions. For obtaining IVD cells, the IVD tissue samples were previously dissected into 2–3 mm^3^ fragments and enzymatically digested for 1 h in 2 mg/mL pronase E (Sigma-Aldrich) in DMEM, under slow stirring, reduced oxygen atmosphere (37°C, 6% O_2_ and 8.5% CO_2_) and saturated humidity. Cells were collected by centrifugation at 400 g for 10 min and washed twice with cold PBS using the same centrifugation settings. For IVD cells only, for more efficient RNA recovery, after the addition of isopropanol, the RNA extraction was carried out using the ReliaPrep RNA Cell Miniprep System (Promega), according to manufacturer's instructions. Total RNA was quantified by Nanodrop (Thermo Fisher). Complementary DNA (cDNA) was obtained through the High-capacity cDNA reverse transcription kit (Applied Biosystems), according to the manufacturer's instructions.

### Quantitative Real-Time Polymerase Chain Reaction (qPCR)

Macrophage gene expression was assessed using TaqMan Gene Expression Master Mix and TaqMan Gene Expression Assays (Applied Biosystems), namely: C-C chemokine receptor type 7 (CCR7): Hs1013469_m1; tumor necrosis factor alpha (TNF-α): Hs00174128_m1; cluster of differentiation 163 (CD163): Hs00174705_m1; matrix metalloproteinase 7 (MMP7): Hs01042796_m1; and glyceraldehyde 3-phosphate dehydrogenase (GAPDH): Hs99999905_m1, as a reference gene.

Regarding IVD gene expression, iQ™ SYBR® Green Supermix (Bio-Rad) was used and the analysis was carried out as before (17). Briefly, specific primer pairs were designed for bovine interleukin 6 (IL-6), IL-8, aggrecan (ACAN), collagen II (COLII), MMP3, and GAPDH and synthesized by Thermo Fisher Scientific.

Quantitative polymerase chain reaction (qPCR) was carried out in an iQ5 Real-Time PCR Detection System (Bio-Rad Laboratories).

Relative gene expression levels were calculated using the quantification cycle (Cq) method, according to Livak and Schmittgen (23). Gene expression levels were presented as 2^−(ΔCt)^, where the average Ct value of each sample was normalized to the house-keeping gene GAPDH [ΔCt = Ct_(geneofinterest)_ - Ct_(GAPDH)_]. Normalized values of samples collected at the end of the experiments were compared with the control and between the different experimental groups.

### Enzyme-Linked Immunosorbent Assay (ELISA)

Culture medium collected at day 13 was centrifuged (3,000 rpm, 5 min) and kept at −20°C for posterior analysis. Human IL-6, TNF-α, IL-8, monocyte chemoattractant protein 1 (MCP-1) and vascular endothelial growth factor (VEGF) were quantified by ELISA (Human Standard TMB ELISA Development Kits, PeproTech) according to manufacturer's instructions. Cytokine and VEGF concentrations (pg/mL) were determined using a standard calibration curve.

### Statistical Analysis

Statistical analysis was performed using GraphPad Prism version 7 (GraphPad Software, Inc.) to evaluate significant differences between the different samples. For macrophage data non-parametric Friedman Test was used, followed by Dunn's multiple comparison test. For IVD data, non-parametric unpaired Kruskal-Wallis test was used followed by Dunn's multiple comparison test. Statistical significance was considered for *p* < 0.05 (^*^p < 0.05, ^**^p < 0.01, ^***^p < 0.001).

## Results

### Macrophages and IVD: Establishment of the Organ Culture

**Figure 2:**
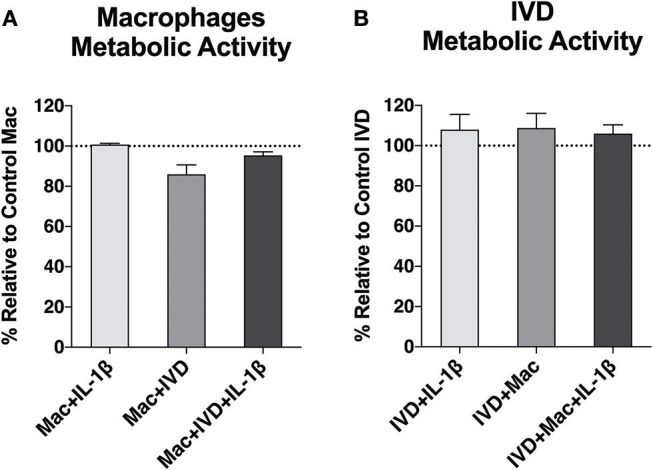
Metabolic activity of macrophages and IVDs, in normal or proinflammatory/degenerative conditions, evaluated using the resazurin assay three days after co-culture. Results are presented as percentage relative to control macrophages **(A)** or control IVDs **(B)** (*n* = 6 macrophage donors and *n* = 4 bovine IVD donors).

First, we investigated whether macrophage viability would be affected by the proinflammatory/degenerative IVD environment and whether IVDs viability would be compromised by the presence of macrophages. Macrophages differentiated from primary monocytes upon 10 days (7 days with M-CSF+3 days without M-CSF) were used. Macrophage differentiation was confirmed by the high level of CD14 expression (see Supplementary Data). Macrophage metabolic activity was assessed through resazurin assay (Figure 2A). The results showed that mitochondrial metabolic activity of macrophages was not affected when IL-1β was added to the media, but slightly decreased when macrophages were co-cultured with IVD in the absence of pro-inflammatory stimuli. We did not observe the same effect in the presence of IVD and IL-1β. IVDs metabolic activity was not altered by the presence of macrophages (Figure 2B).

### Impact of IVD Organ Culture on Macrophage Gene Expression Profile

**Figure 3:**
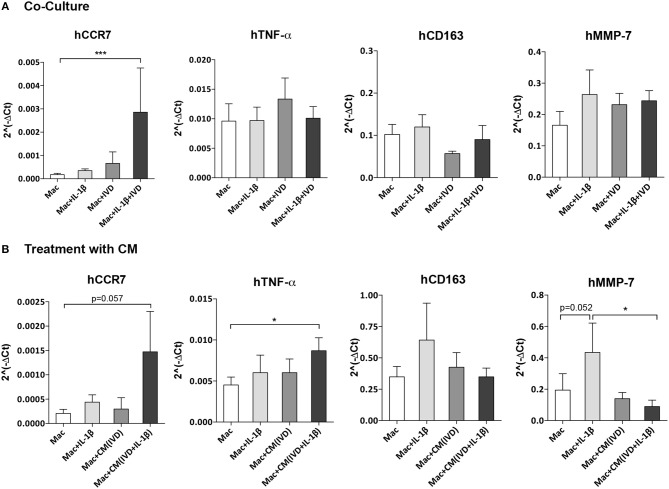
Macrophage gene expression profile alone or in the presence of IVD, in normal or proinflammatory/degenerative conditions. **(A)** Three days after co-culture, macrophage mRNA expression of human CCR7 (hCCR7), hTNF-α, hCD163 and hMMP-7 was evaluated. mRNA levels were normalized to hGAPDH control gene (*n* = 5 macrophage donors and *n* = 3 bovine IVD donors; ***p < 0.001) **(B)** Three days after treatment with IVD conditioned media (CM), macrophage mRNA expression of human CCR7 (hCCR7), hTNF-α, hCD163, and hMMP-7 was evaluated. mRNA levels were normalized to hGAPDH control gene (*n* = 6 macrophage donors and *n* = 3 bovine IVD donors; *p < 0.05).

**Figure 4:**
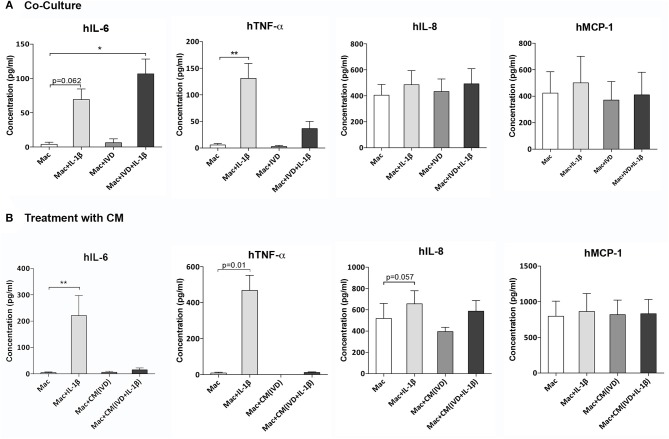
Macrophage cytokine production alone or in the presence of IVD, in normal or proinflammatory/degenerative conditions. **(A)** Three days after co-culture, macrophage cytokine production of human IL-6 (hIL-6), hTNF-α, hIL-8, and hMCP-1 was evaluated by ELISA. Concentration in the culture media presented as pg/mL (*n* = 8 macrophage donors and n=5 bovine IVD donors; *p < 0.05, **p < 0.01) **(B)** Three days after treatment with IVD conditioned media (CM), macrophage cytokine production of human IL-6 (hIL-6), hTNF-α, hIL-8 and hMCP-1 was evaluated by ELISA. Concentration in the culture media presented as pg/mL (*n* = 5 macrophage donors and n=3 bovine IVD donors; **p < 0.01).

Macrophage profile in the presence of IVD organ cultures was evaluated by gene expression of two pro-inflammatory markers (hCCR7 and hTNF-α), one anti-inflammatory marker (hCD163) and one MMP (hMMP-7) after 3 days of co-culture with IVD (Figure 3A). In the presence of the proinflammatory/degenerated IVD organ culture, a significant (*p* < 0.001) upregulation of hCCR7 gene expression in macrophages was observed, that did not occur in basal conditions, in presence of IL-1β or in presence of IVD by itself (Figure 4A). Regarding hTNF-α and hCD163 genes, no statistical difference was observed between the groups (Figure 3A). When macrophages were in the presence of (IVD+IL-1β)-CM, the same tendency of hCCR7 upregulation was observed, however without reaching statistical significance (*p* = 0.057) (Figure 3B). Contrarily to what was observed in the presence of IVD, when macrophages were cultured with CM, hTNF-α gene expression was significantly up-regulated (*p* < 0.01) in presence of (IVD+IL-1β)-CM, reinforcing the differentiation of macrophages toward a more proinflammatory phenotype (Figure 3B). Regarding hMMP-7 gene expression it was up-regulated in IL-1β-treated macrophages compared with basal conditions (Figure 4B, *p* = 0.052) and was significantly down-regulated in presence of (IVD+IL-1β)-CM (*p* < 0.05) compared with IL-1β treatment of macrophages, suggesting that despite IL-1β inducing this MMP gene expression in macrophages, the IVD produces molecules that can inhibit this upregulation (Figure 3B). In addition, surface marker expression for M1 (CD86) and M2 (CD163) markers was evaluated by flow cytometry, however no significant differences were observed between the different conditions tested (see Supplementary Data).

### Impact of IVD Organ Culture on Macrophage Cytokine Production Profile

To further evaluate the modulation of macrophage phenotype by IVD, human cytokine production was analyzed by ELISA, in macrophage co-cultures with IVD (Figure 4A) and with IVD-CM (Figure 4B). As expected, macrophages produced higher levels of hIL-6 (*p* = 0.062), hTNF-α (*p* < 0.01) and hIL-8 (*p* = 0.052) when treated with IL-1β (Figures 4A,B). Control groups with bovine IVD cultures were performed, demonstrating cytokines species specificity. However, when in presence of the proinflammatory/degenerated IVD, hIL-6 levels were significantly higher (*p* < 0.05) (Figure 4A), which was not observed when macrophages were treated with (IVD+IL-1β)-CM, suggesting that the crosstalk between macrophages and IVD is crucial for the production of this pro-inflammatory cytokine (Figure 4B). Surprisingly, the trend observed for increased levels of hTNF-α gene expression in the presence of (IVD+IL-1β)-CM (Figure 3B), was not confirmed at the protein level (Figure 4B), suggesting some post-transcription regulation affecting protein production. Regarding hIL-8 and hMCP-1 no differences were observed in the presence of the different groups or in the presence of IVD-CM (Figures 4A,B).

### Influence of Macrophages on IVD Cells Gene Expression Profile

**Figure 5:**
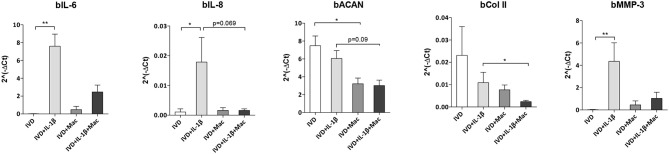
IVD cell gene expression profile alone or in the presence of macrophages, in normal or proinflammatory/degenerative conditions. Three days after co-culture, IVD cell mRNA expression of bovine IL-6 (bIL-6), bIL-8, bACAN, bCOLII and bMMP-3 was evaluated. mRNA levels were normalized to bGAPDH control gene (*n* = 4–12 bovine IVD donors and *n* = 6 macrophage donors; *p < 0.05, **p < 0.01).

The crosstalk between IVD and macrophages was also evaluated by assessing how macrophages influence IVD cells gene expression (Figure 5). Selection of bovine genes was performed based on our previous work (17). Both bIL-6 (*p* < 0.01), bIL-8 (*p* < 0.05) and bMMP-3 (*p* < 0.01) were significantly upregulated in the proinflammatory/degenerated IVD model, while bACAN and bCOLII were reduced, comparatively to the normal IVD (Figure 5), confirming what was previously reported (17). However, when macrophages were added to the proinflammatory/degenerated IVD model, bIL-6, bIL-8, and bMMP-3 were reduced (Figure 5), suggesting that macrophages reduced the pro-inflammatory profile and ECM remodeling proteases by IVD cells in the presence of IL-1β. Regarding the expression of ECM components by IVD cells, macrophages contribute to decrease bACAN (*p* = 0.09) and bCOLII (*p* < 0.05) in proinflammatory/degenerative conditions (Figure 5). bACAN was even down-regulated in IVD in the presence of macrophages in basal conditions (*p* < 0.05) (Figure 5).

### Influence of Macrophages on IVD Production of Angiogenic Factors

**Figure 6:**
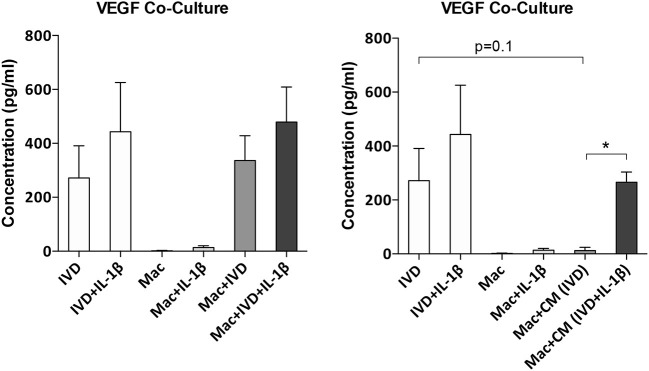
VEGF production by IVD cells or macrophages in co-culture conditions or after macrophage treatment with IVD conditioned media (CM). VEGF production was evaluated by ELISA (*n* = 5–14 bovine IVD donors and *n* = 6 macrophage donors; *p < 0.05).

Increased angiogenesis is one of the phenomena associated with IVD degeneration (24). To evaluate if macrophages can influence the angiogenesis in the IVD microenvironment, the supernatants of macrophages/IVD co-cultures were tested for VEGF presence by ELISA (Figure 6). Our results show that macrophages did not produce VEGF either in basal conditions or in the presence of IL-1β. VEGF is mainly produced by IVD, both in normal or proinflammatory/degenerated conditions, with a slight tendency of increased VEGF production by IVD in the presence of IL-1β (Figure 6). When in co-culture, the presence of macrophages seems to increase VEGF production, however without statistical significance (Figure 6). When macrophages are treated with IVD-CM there is a decrease in VEGF levels, when compared to IVD alone (*p* = 0.1), which is not observed when they are treated with (IVD+IL-1β)-CM where the decrease in VEGF levels is not so accentuated (Figure 6). These observations suggest that macrophages consume more VEGF in normal vs. proinflammatory/degenerated cultured conditions or that in the presence of (IVD+IL-1β)-CM they are consuming VEGF but also producing it. This result indicates that macrophages may act as pro-vascularization mediators within IVD microenvironment.

## Discussion

This study investigates the crosstalk between IVD and macrophages during the process of IVD degeneration. This work is of pivotal importance given the need of adequate models to study the interaction between inflammation and IVD degeneration. Whilst the use of *ex vivo* animal models will not preclude the use of human tissue or *in vivo* models, they may be able to clarify some crucial questions, reducing study costs and ethical concerns (15).

For that purpose, we complexed a proinflammatory/degenerative bovine IVD *ex vivo* model, which had been formerly validated (17), to include human macrophages. These immune cells have been pointed as key players in the process of degeneration-associated pain and hernia resorption (8, 10, 12, 13).

Macrophage mitochondrial function was evaluated using the resazurin reduction assay. Our results evidenced that macrophages and IVD are both metabolically active in co-culture. Metabolic activity slightly decreased when macrophages were in co-culture with IVD in the absence of pro-inflammatory stimuli. This could be a consequence of alterations in macrophage profile. For example, it has been reported that M1 and M2 macrophages exhibit distinct metabolic profiles (25, 26). In M1 macrophages, aerobic glycolysis is induced upon activation, which involves an increase in glucose uptake as well as the conversion of pyruvate to lactate, while M2 macrophages obtained their energy from fatty acid oxidation and oxidative metabolism, which can be sustained for longer periods (25). Thus, M1 macrophages were demonstrated to display enhanced glycolytic metabolism and reduced mitochondrial activity and M2 macrophages show high mitochondrial oxidative phosphorylation (27). Although these metabolic differences between differentially activated macrophages are widely accepted, how the cell's metabolic status regulates polarization and which are the mechanisms responsible for switching the metabolic profile between different phenotypes remains to be understood.

Resazurin is internalized by cells and metabolically reduced to the highly fluorescent pink compound resorufin, that is freely released from cells in a process mediated by intracellular diaphorase enzymes. Rezasurin conversion has been linked to mitochondrial activity, where oxidative phosphorylation occurs. Therefore, our rezasurin results suggest slightly lower levels of oxidative phosphorylation in macrophages cultured with IVD, but not with IVD+IL1β, suggesting a higher level of M2 macrophages in the presence of healthy IVD, which was not confirmed by CD163 expression.

Furthermore, in this proinflammatory/degenerative IVD model, macrophages seem to exhibit a more proinflammatory profile, expressing higher amounts of CCR7 and producing more IL-6. CCR7, a typical macrophage proinflammatory marker, was also upregulated in human samples of degenerated IVD in herniated samples, as reported by Nakazawa and colleagues in non-herniated IVD, in the NP region (13). This is also in accordance with another study showing that macrophages in degenerated IVD samples expressed high levels of iNOS and CD86, two pro-inflammatory markers (12). Macrophages treated with CM from NP cells of degenerated samples have also shown an upregulation of the levels of iNOS (12). Moreover, Takada et al. demonstrated that the levels of IL-6 were increased in a co-culture model of rat IVD and macrophages and that most IL-6 producing cells were macrophages (28). Using the same co-culture model, they found that IVD-macrophage interaction induced an early upregulation of TNF-α, followed by upregulation of IL-6, IL-8, and PGE_2_ (29). We did not observe this increase of TNF-α in our co-culture system and this might be due to the different time points used since they have also described that the levels of this cytokine decreased after 6 h of co-culture (29).

Concerning the influence of macrophages on IVD cell phenotype, our results showed that the levels of IL-6 and IL-8 were increased in IVD cells when they were treated with IL-1β, as showed before (17). Indeed, the levels of these cytokines in disc tissue from patients with LBP were significantly higher than in tissue from patients undergoing discectomy for sciatica (4). Nonetheless, macrophages seem to contribute to a less proinflammatory profile of native IVD cells under proinflammatory/degenerative conditions since there is an apparent decrease, although not statistically significant, of IL-6 and IL-8 in IVD cells when macrophages are added to the system. Interestingly, this effect was observed before using the same system with MSCs. While MSCs demonstrated a more proinflammatory profile in co-culture with the proinflammatory/degenerative IVD model, they also contributed to a less proinflammatory profile of native IVD cells (19).

Concerning MMP-3 levels, they were significantly upregulated in IVD cells in the presence of IL-1β, however this upregulation is not observed when macrophages are added to this system, suggesting that macrophages are impairing the expression of this matrix remodeling agent. Haro et al. observed a marked enhancement of MMP-3 protein and mRNA in chondrocytes after exposure to macrophages in a co-culture model (30). This divergence with our results may be due to the differences between study models (murine vs. bovine/human) and/or the culture stimulation with IL-1β. In another study, Haro et al. concluded that the generation of soluble TNF-α by macrophages was essential for the induction of MMP-3 in disc co-cultures (31). Interestingly, our protein levels of TNF-α produced by macrophages were decreased when they were in the presence of IVD + IL-1β, comparatively to when they were treated with IL-1β alone, which can be linked to the low expression of MMP-3 by IVD cells in the presence of macrophages in proinflammatory/degenerative conditions.

Regarding ECM production by IVD cells, we observed a decrease in the gene expression levels of both ACAN and COLII in the presence of macrophages, suggesting that macrophages in pro-inflammatory conditions contribute to aggravate the loss of native ECM components of healthy IVD.

We also evaluated the levels of VEGF in the supernatants of co-cultures and when macrophages were treated with CM to better understand if macrophages can influence angiogenesis in the IVD microenvironment. Our results demonstrated that this pro-angiogenic growth factor seems to be mainly produced by IVD cells. Nonetheless, the levels of VEGF decrease when macrophages were treated with IVD-CM comparatively to IVD alone, and this accentuated reduction was not observed when they were treated with (IVD+IL-1β)-CM. This result suggests that VEGF can be consumed in higher amounts in healthy comparatively to proinflammatory/degenerative IVD or, that macrophages start to produce VEGF in these conditions, which overall demonstrates that these cells can contribute to a more pro-vascularization microenvironment. However, angiogenesis is a highly complex process, involving several factors. In the future, the evaluation of the expression of other angiogenesis-related factors and an angiogenesis functional assay will be conducted.

This model can be a new tool to address the role of macrophages in IVD degeneration, which is somehow neglected in the literature, although might be limited in the analysis of the immune cell response to a tissue from different species. Nevertheless, the analysis of human macrophage response to bovine IVD tissue in two different scenarios, healthy vs. pro-inflammatory/degenerative conditions, safeguards the conclusions obtained.

Taken together, our results demonstrate a more pro-inflammatory profile of macrophages when they were in presence of proinflammatory/degenerative IVD, which is in concordance with previous findings using human samples (12, 13).

## Conclusions

Overall, the co-culture system established in this study seems to provide a simple and useful model to investigate *in vitro* the interaction between macrophages and IVD. This model can be a valuable tool to characterize the mechanisms by which macrophages and IVD cells interact during IVD aging and degeneration. By constituting a more refined model of the study of inflammation in degenerated IVD, this model may be used for drug screening before animal experimentation and may provide new targets to LBP.

## Data Availability

All datasets generated for this study are included in the manuscript and/or the Supplementary Files.

## Author Contributions

AS, SS, and RG contributed to the study conception and design. AS, JC-R, JF, CC, and MB-G contributed to the acquisition of data. AS and RG contributed to the analysis and interpretation of data and drafted the article. MB, SS, and RG provided the funding for the experiments. All authors have critically revised the article for important intellectual content, and all authors approved the final submitted version.

### Conflict of Interest Statement

The authors declare that the research was conducted in the absence of any commercial or financial relationships that could be construed as a potential conflict of interest.
