# Supplementary material for: Macrophages Down-Regulate Gene Expression of Intervertebral Disc Degenerative Markers Under a Pro-inflammatory Microenvironment
Source: Front Immunol. 2019 Jul 3;10:1508. doi: 10.3389/fimmu.2019.01508 (PMC6616110; doi:10.3389/fimmu.2019.01508)
Supplement: Supplementary file 1 [file Data_Sheet_1.docx]

**Supplementary Data**

Figure S1: Surface marker expression of macrophages upon differentiation with M-CSF (7 days) and before IVD co-culture (day 10). Lineage marker (CD14), M1 marker (CD86) and M2 marker (CD163) were evaluated by flow cytometry (n=4 macrophage donors; *p<0.05).

Figure S2: Surface marker expression of macrophages after 13 days of culture. Lineage marker (CD14), M1 marker (CD86) and M2 marker (CD163) were evaluated by flow cytometry. Evaluation of macrophage surface marker profile alone or in the presence of IVD, in normal or proinflammatory/degenerative conditions, three days after co-culture (n=6 macrophage donors and n=3 bovine IVD donors; *p<0.05)
